# Asthenic Cases

**Published:** 1908-11-28

**Authors:** 


### Mental Diseases.

### ASTHENIC CASES.

Cases are met with in practice which present to the physician a clinical picture of extreme mental and physical fatigue, yet in which the mental symptoms are so pronounced and severe that certification and removal from home become necessary. The condition of the patient resembles, to a certain extent, that seen in low forms of delirium tremens or in the typhoid state, but with mental symptoms more unmanageable and more persistent. These cases probably have a toxic origin, and as a rule follow a more or less prolonged period of ill health, often preceded by influenza.

The physical symptoms are well marked, and are principally those of great nervous fatigue. The skin is earthy in colour and clammy; on pressure, tache cerebrale appears slowly and is persistent. The tongue is hard, dry and brown, much sordes collects on the teeth and lips, and the breath is fœtid. The bowels are as a rule constipated, though there may be diarrhœa; food is taken very badly, and there is often some vomiting after feeding. The temperature varies between normal and 100°, with, perhaps, a sudden evening rise to 102°, which rapidly falls to normal again and does not recur. Lessened muscular tone and power are very noticeable in the loss of facial expression and in the difficulty in swallowing. Muscular tremors and twitchings are present when at rest as well as when movements are made, and fibrillary twitchings are seen. The tongue is markedly tremulous, the speech stumbling and uncertain. Reflexes are exaggerated and there is great apparent inco-ordination of movement. Sensation is dulled more or less, though there is not usually any definite anæsthesia. The heart is affected with the general condition, and easily becomes irregular and feeble in action.

The mental symptoms become rapidly acute before these physical ones are manifest, rendering the management of the patient at home a difficult matter. The earliest symptoms are changes in behaviour and habits, followed by delusions of injury or persecution by certain people. Often the patient believes that relatives and friends are trying to poison him; and it is the violent behaviour due to these delusions which leads to certification.

As the physical symptoms begin to become prominent, the mental ones become more asthenic in character. There is incoherence, inability to answer, perhaps even to understand a simple question, and at times, for prolonged periods, noisiness. A morbid change in the emotions is shown by frequent weeping without apparent cause. Restlessness is a constant symptom, the patient continually pulling off clothes and bed-clothes, and picking at them. Perpetual attempts are made to get out of bed, and if they are successful the patient wanders aimlessly about until he falls. Food is taken badly, forcible feeding being often necessary. Habits become faulty, and the patient will pass everything under him, constant changing of clothes being necessary. There is active resistance to everything that has to be done, and often striking out at those in attendance. There are delusions as to the identity and intentions of friends and attendants, together with a marked antipathy to them. Hallucinations of sight, hearing, and common sensation are present and can be diagnosed from the behaviour. Memory is greatly impaired and remains so until far into convalescence. As regards the attack itself, there is usually no subsequent recollection of any of its incidents.

As was remarked above, all these symptoms point to extreme physical and mental fatigue; but the muscular inco-ordination and the speech may give rise to suspicions of inorganic nervous disease. This inco-ordination of movement and stumbling speech, however, will be found on examination to be due to lack of ability, through weakness, to fix the attention. The patient can usually be roused by gently shaking and speaking sharply to him, and his attention held for a few moments before it rapidly wanders again. During these few moments test movements are made without inco-ordination and test words are clearly pronounced.

Treatment is on general lines, and, if begun early, before physical weakness becomes extreme, recovery generally follows unless there is some pre-existing organic disease to complicate matters. The patient should be placed in a bed made up on the floor, or on a low bedstead against a wall, with a mattress on the floor at the side to prevent injury due to falling. Clothes should be fixed on in some way to prevent their removal and danger from chills. The bowels should be well cleared by enemata at once. The foul state of the mouth accounts for most of the refusal of food, and a mouthwash of chlorine water or Hermitine will do much to relieve the condition. Should the refusal of food persist, and forcible feeding be necessary, the stomach should be first washed out. The liability to bed-sores is very great in these cases, especially as they are invariably "wet." Stimulants are to be avoided unless some special indication for their use arises. In convalescence tonics are valuable.

